# Lipid and immune dysregulation and risk of metabolic disorders after HCV clearance in HIV/HCV-coinfected participants with cACLD: a retrospective study

**DOI:** 10.3389/fimmu.2025.1674837

**Published:** 2026-01-12

**Authors:** Ana Virseda-Berdices, Belen Requena, Juan Berenguer, Juan González-García, Carolina Gonzalez-Riano, Cristina Díez, Victor Hontañon, Amanda Fernández-Rodríguez, Coral Barbas, Salvador Resino, Rubén Martín-Escolano, María Ángeles Jiménez-Sousa

**Affiliations:** 1 Unidad de Infección Viral e Inmunidad, Centro Nacional de Microbiología (CNM), Instituto de Salud Carlos III (ISCIII), Madrid, Spain; 2 Centro de Investigación Biomédica en Red en Enfermedades Infecciosas (CIBERINFEC), Instituto de Salud Carlos III (ISCIII), Madrid, Spain; 3 Centro de Metabolómica y Bioanálisis (CEMBIO), Facultad de Farmacia, Universidad San Pablo-CEU, CEU Universities, Urbanización Montepríncipe, Boadilla del Monte, Spain; 4 Unidad de Enfermedades Infecciosas/VIH; Hospital General Universitario “Gregorio Marañón”, Madrid, Spain; 5 Instituto de Investigación Sanitaria Gregorio Marañón (IiSGM), Madrid, Spain; 6 Servicio de Medicina Interna-Unidad de VIH, Hospital Universitario La Paz, Madrid, Spain; 7 Instituto de Investigación Sanitaria La Paz (IdiPAZ), Madrid, Spain

**Keywords:** HCV clearance, HIV/HCV-coinfection, immunity, lipidomic profile, metabolic events

## Abstract

**Introduction:**

People with HIV and chronic hepatitis C may develop metabolic complications after sustained virologic response (SVR), possibly due to persistent molecular alterations induced by HCV. This study aimed to identify baseline (pre-treatment) lipid and immune biomarkers associated with post-SVR metabolic events in HIV/HCV-coinfected participants with compensated advanced chronic liver disease (cACLD) receiving long-term suppressive antiretroviral therapy.

**Methods:**

We conducted a retrospective study of 56 HIV/HCV-coinfected participants with cACLD. Untargeted lipidomic profiling was performed on baseline plasma samples using a liquid-chromatography-mass spectrometer. The outcome was the development of metabolic events (diabetes mellitus and/or hyperlipidemia) during follow-up, up to seven years post-HCV treatment. Statistical analyses included orthogonal partial least squares discriminant analysis (OPLS-DA), Cox regressions models, and Spearman correlations with inflammation-related biomarkers and immune checkpoint proteins, with multiple comparison corrections using the false discovery rate.

**Results:**

25% participants developed metabolic events after SVR. OPLS-DA identified 163 lipid species (VIP scores≥1) associated with these events, and adjusted Cox regression confirmed significant associations for 24 of them. Lysophosphatidylcholines (LPCs) were the most prevalent, with higher baseline levels linked to increased metabolic risk. Participants who developed events also had higher levels of plasmalogens LPC (O-LPC), lysophosphatidylethanolamine (LPE), lysophosphatidylinositol (LPI), lysophosphatidic acid (LPA), and phosphosphatidylcholine (PC). Several lipid species correlated positively with the pro-inflammatory cytokine IL-18, the anti-inflammatory suppressor IL-1RA, and the immune checkpoint proteins IDO and S100A8/A9.

**Discussion:**

Pre-treatment lipid and immune dysregulation was associated with post-SVR metabolic events in HIV/HCV-coinfected participants, suggesting that HCV may leave a lasting metabolic imprint that contributes to adverse outcomes after viral clearance.

## 1 Introduction

The chronic hepatitis C virus (HCV) has been associated with a wide range of metabolic complications. HCV replication is closely linked to increased lipid biosynthesis and decreased lipid degradation, contributing to hepatic steatosis (1) and the development of Metabolic Associated Fatty Liver Disease (MAFLD) (2), type 2 diabetes mellitus (T2DM), and/or hyperlipidemia (3). These metabolic alterations can also play a significant role in liver disease progression (4) and may increase the risk of cardiovascular events, cancer, and mortality (5–8).

Coinfection with human immunodeficiency virus (HIV) increases HCV viremia (9), leading to more rapid HCV-associated disease progression than HCV monoinfection (10). In addition, HIV/HCV-coinfected individuals are at increased risk of metabolic complications, mainly due to HIV-mediated effects and exposure to antiretroviral therapy (ART) (11). Importantly, the likelihood of such complications is further elevated in the context of advanced fibrosis or cirrhosis (12–16). To better capture the continuum between advanced fibrosis and cirrhosis in asymptomatic individuals—where clinical differentiation is often unfeasible—the term compensated advanced chronic liver disease (cACLD) has been proposed. This concept underscores the progressive nature of liver damage and the associated risk of clinically significant portal hypertension (17).

The impact of successful HCV therapy on the progression of HCV-related metabolic complications has been widely discussed (18), particularly in people with HIV (PWH) (10, 19–21). Even after achieving sustained virologic response (SVR), some patients remain at risk of developing metabolic syndrome, one of the hallmarks of chronic hepatitis C (22). In this regard, persistent molecular changes induced by chronic HCV infection and associated with the risk of severe disease could explain why HCV cure only partially reduces this risk (23), particularly in HIV/HCV-coinfected patients (20).

In the era of curative HCV therapy, identifying predictive biomarkers is essential for more closely monitoring patients who remain at risk of developing metabolic complications. Therefore, a deeper understanding of the pathophysiological mechanisms involved in the development of metabolic disorders among these patients is crucial. Thus, in this study, we aimed to evaluate plasma lipids and immune markers at baseline (before HCV therapy) associated with metabolic events occurring after successful HCV treatment in HIV/HCV-coinfected participants on long-term suppressive ART with cACLD.

## 2 Materials and methods

### 2.1 Study subjects

We carried out a multicenter retrospective study in 56 HIV/HCV-coinfected participants with cACLD on long-term suppressive ART from 10 centers in Spain (see **Supplementary Data 1**). cACLD was defined according to the Baveno VI consensus conference as a LSM ≥10 kPa or liver biopsy showing bridging fibrosis or cirrhosis (17).

These participants had started therapy with direct-acting antivirals (DAAs) or IFN-based (peg-IFN-α/ribavirin or peg-IFN-α/ribavirin/DAAs) therapy between February 2012 and September 2015, achieving SVR (undetectable HCV-RNA load 12–24 weeks – depending on regimen – after the finalization of anti-HCV treatment). All participants had available clinical data and samples of frozen plasma at the start of HCV treatment (baseline). The end of follow-up was between January 2019 and May 2021. All participants were on stable ART for over six months and had an undetectable plasma HIV viral load (<50 copies/mL). Participants with hepatitis B virus (HBV) coinfection, acute hepatitis C, hepatocellular carcinoma, hepatic decompensation, or a history of metabolic events (including those occurring before completion of HCV therapy) were excluded from the study.

The study was approved by the Research Ethics Committee of the Institute of Health Carlos III (CEI PI 72_2021) and was carried out according to the Declaration of Helsinki. Before registration, all participants signed a written consent.

### 2.2 Clinical data and samples

Epidemiological, clinical, and virological characteristics of participants were collected from medical records using an online form, meeting all confidentiality requirements. This information was monitored.

Peripheral blood samples were collected in EDTA tubes and stored at -80°C in the HIV HGM BioBank (http://hivhgmbiobank.com/?lang=en) until use.

### 2.3 Outcome variable

The outcome was the occurrence of a metabolic event (dichotomous) defined as T2DM and/or hyperlipidemia developed during the follow-up. T2DM was defined as symptoms of diabetes plus casual plasma glucose concentration ≥ 200 mg/dL, fasting plasma glucose concentration ≥ 126 mg/dL, or 2-hour plasma glucose ≥ 200 mg/dL during an oral glucose tolerance test (24). Hyperlipidemia was defined as total cholesterol ≥ 200 mg/dL, low-density lipoprotein (LDL) cholesterol ≥ 130 mg/dL, and/or serum triglycerides ≥ 150 mg/dL (25).

### 2.4 Non-targeted lipidomics analysis

The list of reagents and standards, metabolite extraction, quality management assurance, analytical conditions, and metabolite annotation are available in more detail in **Supplementary Data 2**.

#### 2.4.1 Lipid extraction

Inactivated plasma samples were processed following an extraction protocol consisting of MeOH: MTBE (1:1, v/v) at the Centro de Metabolómica y Bioanálisis, CEMBIO (Madrid, Spain).

#### 2.4.2 Quality management assurance and blank samples

Quality Control (QC) samples were prepared by pooling equal volumes of each plasma sample. These QC samples were then processed in parallel with the rest of the experimental samples in the same manner. Along with the other samples, two blank samples were prepared using the same lipid extraction procedure with the sample solvents. They were injected at the beginning and end of the analytical sequence to find common contaminations.

#### 2.4.3 Analytical conditions

We performed an untargeted lipidomics analysis to cover the broader spectrum of the plasma lipidome. Samples were analyzed using an Agilent 1290 Infinity II Ultra-High-Performance Liquid Chromatography (UHPLC) system coupled to an Agilent 6546 Quadrupole Time-of-Flight (QTOF) Mass Spectrometer (MS) equipped with dual Agilent Jet Stream (AJS) Electrospray (ESI) ion source. Data were collected in separate runs in positive and negative ESI modes.

At the end of the analysis, iterative MS/MS acquisition mode was performed for both ionization modes using QC samples. The MS/MS information for most of the plasma lipidome was obtained by collecting thousands of MS/MS spectra using MassHunter Workstation Software LC-MS Data Acquisition v B.09.00 (Agilent Technologies, Waldbronn, Germany).

#### 2.4.4 Lipid annotation process

The lipid annotation process consisted of using a combination of four bioinformatics tools based on three different annotation strategies (26): spectra matching (Lipid Annotator, MS-Dial), the bottom-up strategy (LipidHunter), and the fragment intensity rules (LipidMS). The complete description of the software parameters can be found in **Supplementary Data 2**. This comprehensive approach, using multiple tools, led to a significant improvement in the lipid annotation process.

Subsequently, redundant data and false positive annotations were eliminated by combining and manually curating the collected information. In addition, the tentative annotation provided by the software annotation tools was combined with the manual inspection of the MS and MS/MS spectra data of the samples, based on the fundamentals of structural elucidation and the assistance of the CEU Mass Mediator (CMM) (27) to corroborate the accuracy of the lipid annotations.

### 2.5 Data reprocessing

The raw data files were then imported into the Agilent MassHunter Profinder software (B.10.0.2, Agilent Technologies, Santa Clara, CA, USA) to be reprocessed employing the already elaborated list of lipids and using the “Batch Targeted Feature Extraction” mode for time alignment and feature extraction.

Lipidomics data normalization was conducted utilizing the Kuligowski transformation (28) implemented in MATLAB (R2023a, MathWorks). Lipid species were selected based on their coefficient of variation (CV) within the QC samples, applying a threshold of 30%. Further details about data reprocessing and normalization can be found in **Supplementary Data 2**.

### 2.6 Multiplex immunoassays and ELISA

ProcartaPlex™ multiplex (Invitrogen™) panels were used to measure anti-inflammatory/suppressor markers (interleukin-1 receptor antagonist (IL-1RA)), pro-inflammatory cytokines markers (IL-18), pro-inflammatory chemokines (interferon-inducible protein 10 (IP-10) and monocyte chemoattractant protein-1 (MCP-1)), and endothelial dysfunction markers (tumor necrosis factor receptor-1 (TNF-RI)), as well as several immune checkpoint proteins (ICPs) (B and T lymphocyte attenuator (BTLA), cluster of differentiation 137 (CD137), CD152(CTLA4), CD27, CD28, glucocorticoid-induced TNFR-related (GITR), herpesvirus entry mediator (HVEM), indoleamine 2,3-dioxygenase (IDO), lymphocyte activation gene-3 (LAG-3), programmed cell death protein 1 (PD-1), programmed death-ligand 1 (PD-L1), programmed death-ligand 2 (PD-L2), T-cell immunoglobulin and mucin-domain containing-3 (TIM-3), B7-H6, CD134(OX40), CD276(B7-H7), CD47 (integrin-associated protein, IAP), CD48 (B-lymphocyte activation marker, BLAST-1), ICOS Ligand (B7-H2), S100A8/A9, T-cell immunoglobulin and mucin domain containing 4 (TIMD-4) and VISTA (B7-H5)). Raw fluorescence intensity (FI) values (arbitrary units, a.u.) were used.

### 2.7 Statistical analysis

For the group description, continuous variables were expressed as median (interquartile range, IQR), and categorical variables as absolute count (percentage). The Mann–Whitney U and Chi-square tests were used to compare independent groups for continuous and categorical variables, respectively.

For the multivariate lipidomic analysis, data were log-transformed (log10) and auto-scaled. The multivariate lipidomic analysis was carried out using MetaboAnalyst 5.0 software (http://www.metaboanalyst.ca/). Orthogonal partial least squares discriminant analysis (OPLS-DA) was performed, and variable importance in projection (VIP) values were obtained.

For univariate regression analysis, those plasma lipids with VIP > 1 were subjected to Cox regression models to analyze the association between individual plasma lipids and metabolic events during the follow-up. Firstly, outliers were treated according to the commonly used 1.5×IQR rule: 75th percentile + 1.5 × IQR, and 25th percentile −1.5 × IQR. Cox regression model provides the hazard ratio (HR), the 95% confidence interval (95% CI), and its level of significance (p-value), which was corrected for multiple testing using the False Discovery Rate (FDR) with Benjamini and Hochberg procedure (q-value). Besides, this test was adjusted for the main available epidemiological and clinical characteristics (age, gender, body mass index (BMI), HCV treatment (IFN-based therapy or DAAs), hepatic steatosis index (HSI) at baseline, and HCV viral load at baseline). These covariates were previously selected by a stepwise method (forward), according to the specific model’s lowest Akaike information criteria (AIC). The level of significance was defined as p-value <0.050 (two-tailed) and q-value <0.200.

Finally, the Spearman correlation was carried out to investigate the relationship between significant metabolic event-associated metabolites and ICPs and inflammatory biomarkers. Those suitable correlations (r>0.30 or r<-0.30) with a significant value (p<0.050; q-value<0.200) were considered relevant.

### 2.8 Lipid network

The Lipid Network Explorer platform (LINEX) (29) was used to visualize and analyze the functional associations of lipids that were significantly associated with metabolic events in networks, providing a comprehensive overview of lipid species metabolism. The parameter used to quantify the effect of differential lipid abundance and visualize the differential patterns (increase or decrease in lipid levels in the presence of metabolic events) was the logarithm of fold change (LFC).

## 3 Results

### 3.1 Participants’ characteristics

The baseline characteristics of 56 HIV/HCV-coinfected participants are shown in **Table 1**. Overall, 44 (78.6%) were male, 31 (56.4%) were current smokers, and 22 (40.0%) and 43 (76.8%) had a prior history of alcohol intake and injection drug use, respectively. The median age was 51, and the body mass index (BMI) was 24.6 kg/m2. Regarding the previous virological aspects, 43 (76.8%) participants were infected with HCV genotype 1.

**Table 1:** Baseline clinical, epidemiological, and virological characteristics of HIV/HCV-coinfected participants according to the development of metabolic events during the follow-up.

Variables	All participants	Participants with metabolic event	Participants with no metabolic event	p
**No.**	56	14 (25.0%)	42 (75.0%)	
Age (years)	51 (48–53)	50 (47–53)	51 (49–54)	0.292
Gender (male)	44 (78.6%)	11 (78.6%)	33 (78.6%)	0.999
BMI (kg/m2)	24.6 (21.9–27.1)	25.5 (23.9–28.7)	24.2 (21.2–26.4)	0.080
Smoker (n = 55)	31 (56.4%)	8 (57.1%)	23 (56.1%)	0.999
Alcohol intake (>50g/day) (n = 55)				0.581
Never	30 (54.5%)	8 (57.1%)	22 (53.7%)	
Previous (>6 months)	22 (40.0%)	6 (42.9%)	16 (39.0%)	
Current	3 (5.5%)	0 (0.0%)	3 (7.3%)	
Intravenous drug user				0.999
Never	13 (23.2%)	3 (21.4%)	10 (23.8%)	
Previous (>6 months)	43 (76.8%)	11 (78.6%)	32 (76.2%)	
Current	0 (0%)			
Previous HCV therapy	31 (55.4%)	5 (35.7%)	26 (61.9%)	0.162
Liver markers				
HSI (n = 55)	34.2 (29.2–37.7)	36.5 (34.4–40.7)	33.8 (28.3–37.2)	**0.021**
LSM (kPa)	21.7 (13.4–34.2)	21.0 (11.0–27.3)	23.3 (14.1–35.0)	0.389
APRI	1.7 (0.8–3.2)	1.1 (0.6–2.5)	1.8 (0.9–3.4)	0.315
TyG (n = 54)	8.7 (8.3–9.0)	8.7 (8.3–8.9)	8.7 (8.4–9.0)	0.810
TyG-HDL (n = 50)	2.8 (1.6–6.1)	2.0 (1.2–4.1)	3.1 (1.9–6.1)	0.285
METS-IR (n = 49)	2.5 (2.3–2.7)	2.4 (2.2–2.6)	2.5 (2.3–2.7)	0.428
High blood pressure	8 (14.3%)	4 (28.6%)	4 (9.5%)	0.186
Previous HCV markers				
HCV genotype				0.850
1	43 (76.8%)	10 (71.4%)	33 (78.6%)	
3	8 (14.3%)	2 (14.3%)	6 (14.3%)	
4	4 (7.1%)	2 (14.3%)	2 (4.8%)	
Others	1 (1.8%)	0 (0.0%)	1 (2.4%)	
Log10 HCV-RNA (IU/mL) (n = 55)	6.3 (5.8–6.7)	6.1 (5.6–6.3)	6.5 (6.0–6.7)	**0.035**
HCV-RNA > 850.000 IU/mL)	39 (69.6%)	8 (57.1%)	31 (73.8%)	0.401
HCV therapy				0.334
IFN-based	36 (64.3%)	11 (78.6%)	25 (59.5%)	
DAAs	20 (35.7%)	3 (21.4%)	17 (40.5%)	
HIV markers				
Previous AIDS (n = 55)	1 (1.8%)	0 (0.0%)	1 (2.4%)	0.999
Nadir CD4+/mm3 (n = 54)	156 (85–260)	100 (51–255)	166 (102–260)	0.282
Nadir < 200 CD4+/mm3 (n = 55)	34 (61.8%)	8 (57.1%)	26 (63.4%)	0.922
Baseline CD4+ T-cells/mm3	464 (293–673)	429 (349–606)	487 (262–711)	0.820
Baseline < 500 CD4+/mm3	30 (53.6%)	8 (57.1%)	22 (2.40%)	0.999
HIV antiretroviral therapy (n = 51)				0.070
NRTI + NNRTI	18 (35.3%)	6 (50.0%)	12 (30.8%)	
NRTI + II	21 (41.2%)	1 (8.3%)	20 (51.3%)	
NRTI + PI	8 (15.7%)	4 (33.3%)	4 (10.3%)	
Others	4 (7.8%)	1 (8.3%)	3 (7.7%)	

Statistics: The values are expressed as the absolute number (percentage) and median (interquartile range). P-values were calculated by the Chi-square test and the Mann-Whitney U test.

BMI, body mass index; HSI, hepatic steatosis index; LSM, liver stiffness measurement; kPa, kilopascal; APRI, AST to Platelet Ratio Index; TyG, Triglycerides and glucose index; HDL, High-density lipoprotein cholesterol; METS-IR, metabolic score for insulin resistance; HCV, hepatitis C virus; IU, international units; IFN, interferon; DAAs, direct-acting antivirals; HCV-RNA, viral load of hepatitis C; AIDS, acquired immune deficiency syndrome; NRTI, nucleoside analogue HIV reverse transcriptase inhibitor; NNRTI, non-nucleoside analogue HIV reverse transcriptase inhibitor; II, HIV integrase inhibitor; PI, HIV protease inhibitor.

Statistically significant differences are shown in bold.

During a median follow-up period of 6.09 years (IQR = 5.69–6.48), 14 participants (25.0%) developed metabolic events, with a median time to onset of 1.87 years (IQR = 1.41–2.87), including 2 cases of T2DM and 12 cases of hyperlipidemia. Both participant groups exhibited similar characteristics, except for hepatic steatosis index (HSI) (p-value = 0.021; **Table 1**) and HCV viral load (p-value = 0.035; **Table 1**).

### 3.2 Lipidome detection results

**Figure 1:**
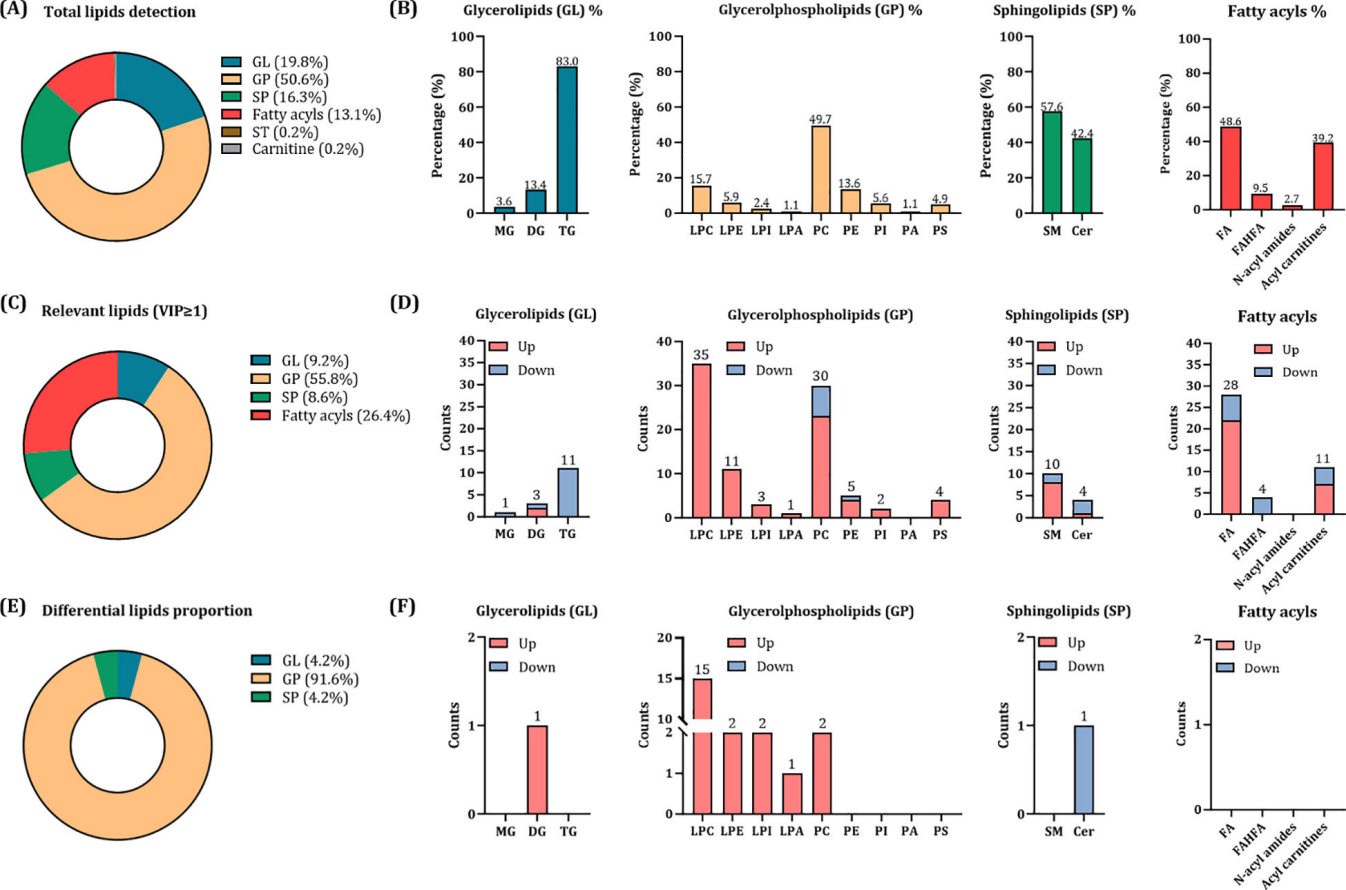
Lipidomic analysis in HIV/HCV-coinfected participants according to the development of metabolic events. **(A)** Proportion of main classes of detected lipids in the analysis. **(B)** Proportion of detected lipid subclasses in each main class. **(C)** Proportion of the main classes of relevant lipids (with a VIP score ≥1). **(D)** Counts of up- and down-regulated relevant lipids (with a VIP score ≥1). **(E)** Proportion of main lipid classes significantly associated with the development of metabolic events. **(F)** Counts of up- and down-regulated differential lipids significantly associated with metabolic events at the subclass level. Glycerolipids (GL) are referred to monoglycerides (MG), diglyceride (DG) and triglyceride (TG); glycerolphospholipids (GP) to lysophosphatidylcholines (LPC), lysophosphatidylethanolamines (LPE), lysophosphatidylinositols (LPI), Lysophosphatidic acids (LPA), phosphatidylcholines (PC), phosphatidylethanolamines (PE), phosphatidylinositols (PI), phosphatidylserines (PS) and phosphatidic acids (PA); sphingolipids (SP) to sphingomyelins (SM) and ceramides (Cer); and fatty acyls to fatty acids (FA), fatty acyl ester of hydroxy fatty acid (FAHFA), N-acyl amides and Acyl carnitines.

After MS data reprocessing, 480 and 374 features were obtained in ESI (+) and ESI (–), respectively. After data combination, normalization, and filtration, a total of 566 distinct plasma lipid species were detected (440 for LC-MS ESI (+) and 126 for LC-MS ESI (–)). According to the LIPID MAPS structure database (LMSD), four main categories of identified lipids were found: 112 glycerolipids (GL, 19.8%), 286 glycerolphospholipids (GP, 50.6%), 92 sphingolipids (SP, 16.3%), 74 fatty acyls (13.1%), along with two additional species (1 sterol lipid (ST, 0.2%) and 1 carnitine (0.2%)) (**Figure 1A**). These main categories were further divided into 17 subclasses, with triglycerides (TG), phosphatidylcholines (PC), sphingomyelins (SM), and fatty acids (FA) having the highest relative abundance, respectively (**Figure 1B**).

### 3.3 Baseline lipid profile associated with the occurrence of metabolic events

**Figure 2:**
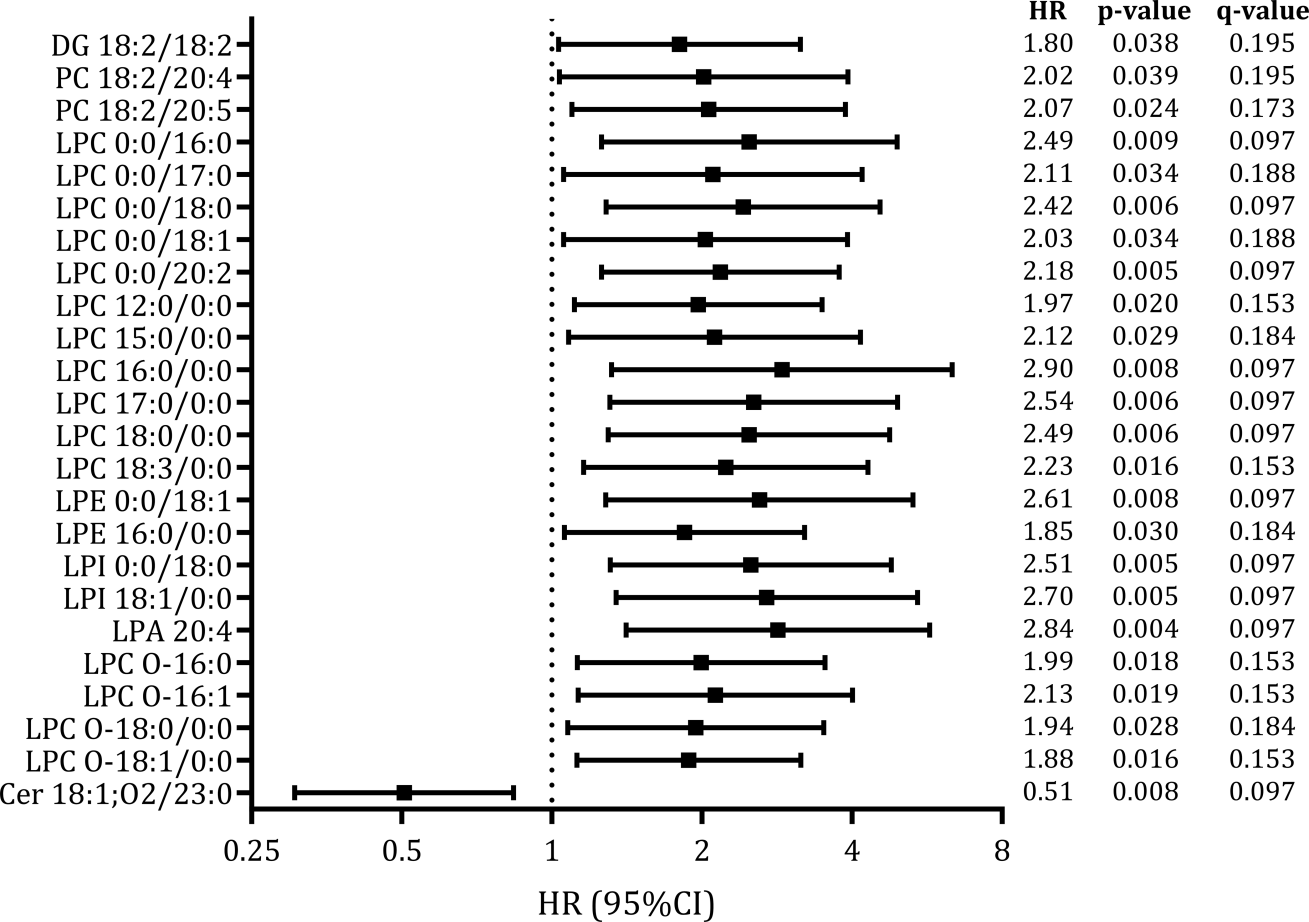
Association of significant baseline plasma lipids with the development of metabolic events years after successful HCV treatment in HIV/HCV-coinfected participants. Statistics: Data were calculated by Cox regression models adjusted by age, gender, body mass index (BMI), HCV treatment (IFN-based therapy or DAAs), hepatic steatosis index (HSI) at baseline, HCV viral load at baseline, and time from baseline to metabolic event or the end of follow-up, using a stepwise model (see Materials and Methods Section). The q-values represent p-values corrected for multiple testing using the False Discovery Rate (FDR). Abbreviations: DG, diglyceride; PC, phosphatidylcholine; LPC, lysophosphatidylcholine; LPE, lysophosphatidylethanolamine; LPI, lysophosphatidylinositol; LPA, lysophosphatidic acid; Cer, Ceramide; HR, hazard ratio; 95%CI, 95% of confidence interval; p, level of significance; q, corrected level of significance.

The OPLS-DA models showed that lipid profiles separated participants according to the development of metabolic events during the follow-up (**Supplementary Figure 1**), identifying a total of 163 plasma lipids as most notably related to metabolic events (VIP scores greater than 1). The distribution of these key discriminant lipids is shown in **Figure 1C**: 55.8% were GP, 26.4% fatty acyls, 9.2% GL, and 8.6% SP. An analysis of their regulation patterns revealed that most of these lipids were upregulated in participants who later developed a metabolic event (**Figure 1D**). Of them, 24 lipid species showed significant associations (p-value <0.050, q-value <0.200) in the adjusted Cox regression models (**Figures 1E-F**; **Figure 2**; **Supplementary Table 1**). GP, mainly lysophosphatidylcholines (LPC), were the most prevalent differential lipids, all displaying higher baseline levels in participants who subsequently developed a metabolic event after HCV clearance. In contrast, GL and SP were less represented, with only DG 18:2/18:2 and Cer 18:1;O2/23:0, showing significant associations.

To further illustrate these differences, the intensity distributions of the most highly significant lipid species (p-value <0.010, q-value <0.100) are presented in **Supplementary Figure 2**. This visualization confirms the elevated baseline abundance of LPC, LPE, LPI, and LPA species, and the reduced abundance of Cer 18:1;O2/23:0, in the group that subsequently developed metabolic events.

### 3.4 Lipid network

**Figure 3:**
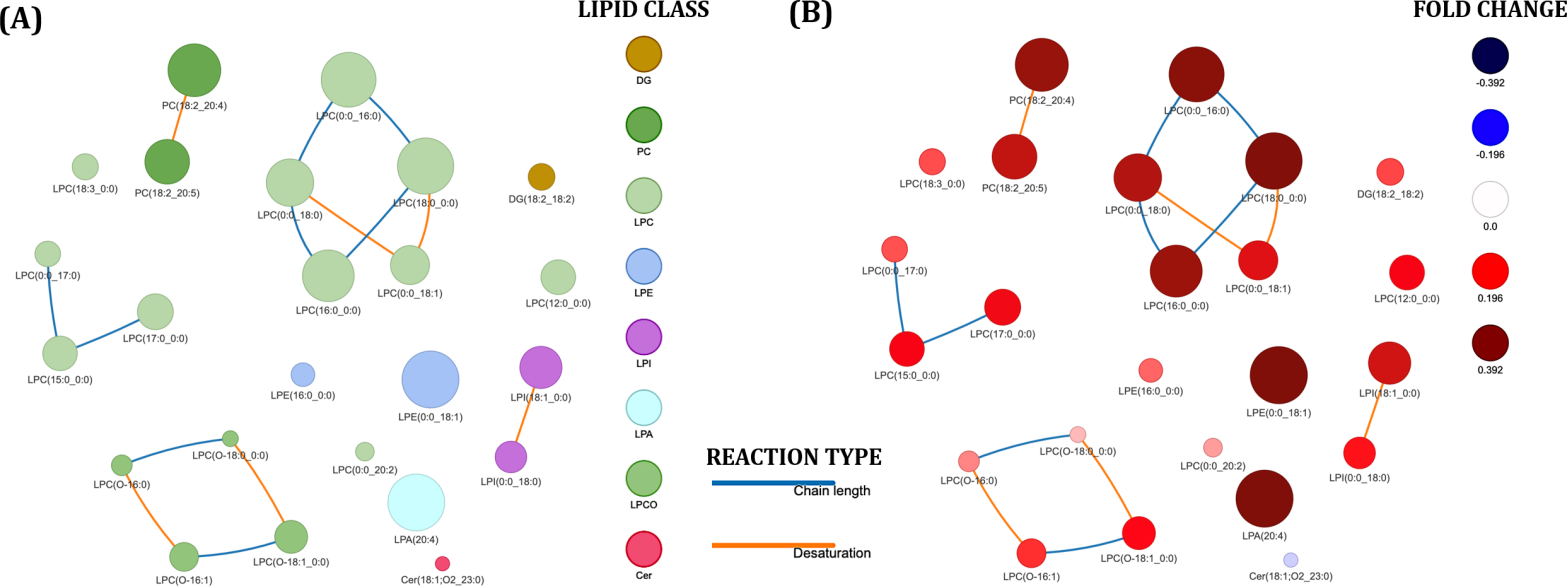
The lipid networks of lipids associated with the occurrence of metabolic events years after successful HCV treatment in HIV/HCV-coinfected participants. Each node represents a lipid species, and edges indicate biochemical reactions that interconvert lipids. Node colors represent the lipid class in **(A)** and the fold change in **(B)**, with red indicating increased and blue indicating decreased lipid levels in participants with metabolic events. Node sizes represent the log fold change between the two conditions, where more strongly altered lipids are displayed as larger nodes.

Global lipid networks for the lipid species significantly associated with metabolic events during follow-up can be visualized in **Figure 3**. Most of the network corresponded to LPCs and plasmalogens LPC species interconnected by changes in the chain length and desaturation (**Figure 3A**). **Figure 3B** provides a visual representation of quantitative lipid level differences between participants with and without metabolic events during the follow-up.

### 3.5 Correlation analysis between significant lipids and inflammatory biomarkers/ICPs

**Figure 4:**
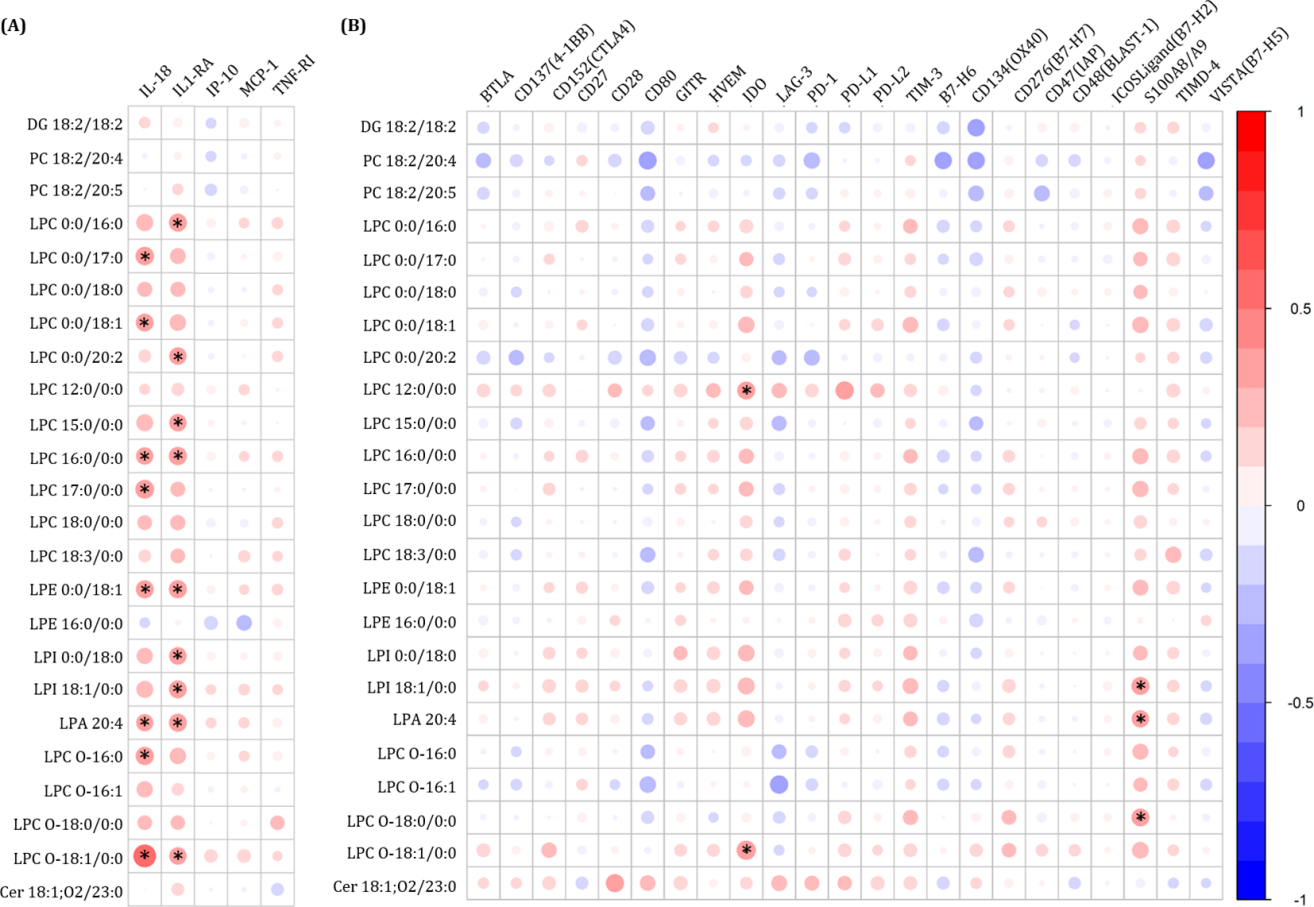
Spearman correlation plot between significant plasma lipids and plasma **(A)** inflammatory-related biomarkers and **(B)** immune checkpoint proteins. The size of the circles is proportional to the strength of the correlation. The color represents the direction (color legends are shown on the right), whereas large dark blue represents a strong negative correlation. A large dark red circle represents a strong positive correlation. Lipids are on the vertical axis, and inflammatory-related biomarkers and immune checkpoint proteins are on the horizontal axis. Those correlations with rho>0.3 o rho<-0.3, p-value<0.05, and q-value<0.2 are shown with an asterisk. DG, diglyceride; PC, phosphatidylcholine; LPC, lysophosphatidylcholine; LPE, lysophosphatidylethanolamine; LPI, lysophosphatidylinositol; LPA, lysophosphatidic acid; Cer, Ceramide; IL, interleukin; IL-1RA, IL-1 receptor antagonist; IP-10, human interferon-inducible protein 10; MCP-1, monocyte chemoattractant protein-1; TNF-RI, tumor necrosis factor receptor-1; BTLA, B, and T lymphocyte attenuator; CD, cluster of differentiation; GITR, glucocorticoid-induced TNFR-related; HVEM, herpesvirus entry mediator; IDO, indoleamine 2,3-dioxygenase; LAG-3, lymphocyte activation gene-3; PD-1, programmed cell death protein 1; PD-L1, programmed death-ligand 1; PD-L2, programmed death-ligand 2; TIM-3, T-cell immunoglobulin and mucin-domain containing-3; TIMD-4, T-cell immunoglobulin and mucin domain-containing protein 4; VISTA(B7-H5), V-domain Ig suppressor of T cell activation.

The pro-inflammatory cytokine IL-18 and the anti-inflammatory suppressor IL-1RA were positively associated with several LPCs, LPE, LPI, and LPA (**Figure 4A**). Regarding ICPs, IDO was positively associated with LPC 12:0/0:0 and LPC O-18:1/0:0, while S100A8/A9 was associated with LPI 18:1/0:0, LPA 20:4, and LPC O-18:0/0:0 (**Figure 4B**).

We also evaluated the predictive value of baseline inflammatory biomarkers for metabolic events using univariate Cox regression analysis. Unlike the lipidomic profile, none of the immune markers reached statistical significance (p-value <0.050, q-value <0.200), although TNF-RI showed a marginal trend (HR = 3.83; p-value =0.075, q-value =0.286). This finding suggests that the identified lipid signature provides a more robust stratification of long-term metabolic risk in this cohort compared to classical immune markers.

## 4 Discussion

In this study, we identified a baseline lipid profile, measured before HCV treatment, that is associated with the occurrence of metabolic events (T2DM and/or hyperlipidemia) years after successful HCV treatment in HIV/HCV-coinfected participants on long-term suppressive ART with cACLD. The majority of significant lipids belonged to the GP class, particularly LPC, LPE, LPI, LPA, and PC, all of which showed increased baseline levels in participants who later developed metabolic events. In contrast, GL and SP were less represented. Several lipid species correlated positively with the pro-inflammatory cytokine IL-18, the anti-inflammatory suppressor IL-1RA, and the immune checkpoint proteins IDO and S100A8/A9. To our knowledge, this is the first long-term follow-up study evaluating the association of baseline plasma lipid and immune marker levels with metabolic events in HIV/HCV-coinfected participants after successful HCV treatment.

Plasma lipid alterations are commonly present in both infected and uninfected individuals with metabolic disorders, such as atherosclerosis and T2DM (30–33). A recent study involving both HIV-uninfected and HIV-infected individuals identified 16 lipid classes associated with T2DM risk (34). Among them, many GL and GP were positively related to the risk of T2DM, which is consistent with our findings. Furthermore, large-scale prospective cohort studies in uninfected European and Asian individuals have demonstrated a positive association between GL and GP levels and the future development of T2DM. These findings suggest their potential as prognostic biomarkers for T2DM, as they significantly improve the risk prediction of T2DM compared to traditional risk factors (32, 35–37). Moreover, elevated levels of GP and GL have been previously reported in hyperlipidemia (38), where their accumulation contributes to lipid dysregulation and the pathogenesis of this metabolic disorder.

GPs are key components in cell membranes and serve as a source of physiologically active compounds that function as signaling molecules. In our study, LPC was the most differentially represented subclass in those participants who later developed metabolic events, with 15 out of 24 lipid species significantly associated with metabolic events belonging to this group. LPCs have been widely reported to be elevated in atherosclerosis (39), inflammatory disease (40), hyperlipidemia (41), and T2DM (41, 42), among others. In addition, high levels of LPCs have been linked to T2DM-related complications (43), such as retinopathy and neurodegeneration (42, 44). Specifically, LPCs can exert their biological effect by inducing cell division, apoptosis, oxidative stress, and the release of inflammatory factors. Through G protein-coupled receptor (GPCR) (LPC receptor) and caspase-1, LPCs can activate biologically inactive pro-cytokines, such as IL-18 (45, 46). In addition, LPCs can enhance chemokine production, facilitating cell recruitment and amplifying inflammatory responses (47). These two pathways play an important role in inflammation and disease pathogenesis. Additionally, participants who later developed metabolic events had increased levels of plasmalogen LPCs, which may have contributed to the occurrence of hyperlipidemia and/or T2DM by exacerbating oxidative stress (48), a known factor implicated in the onset and development of metabolic dysfunctions (49).

Furthermore, five out of the 24 lipid species associated with metabolic events in our study were lysophospholipids LPE, LPI, and LPA. Several studies have found increased levels of LPEs (mainly LPE 16:0) (50) and LPIs (51) in participants with T2DM and have been suggested as biomarkers for predicting T2DM. Collectively, these studies highlight the role of these plasma lipids as independent predictors of T2DM, as well as their association with oxidative stress, inflammation, and mitochondrial dysfunction (51). Structurally, LPEs share similarities with LPCs, differing only in their ethanolamine headgroup; however, their physiological role in T2DM remains unclear. LPIs (inositol headgroup) are involved in numerous physiological actions that are closely related to adipose tissue, including angiogenesis, apoptosis, and inflammation (52, 53). Some studies have indicated that the LPI/GPR55 system is a novel target in obesity (54), and it has recently been suggested to be involved in T2DM development (51). Still, additional studies are required to investigate its potential role. Further, it is important to note that some studies have reported conflicting results, with certain lipids showing negative associations with T2DM risk over time (55–57). However, these discrepancies could be attributed to differences in study design, participant characteristics, or analytical methodologies. Additionally, LPA species have been reported to disrupt hepatic cholesterol homeostasis, showing a significant association with the clinical levels of total cholesterol and LDL-C (58). Besides, LPA has been implicated in impairments of glucose metabolism (59).

Additionally, although GL and SP categories were less represented among the significant lipids (with only one diglyceride and one ceramide, respectively), the broader set of relevant lipids with VIP>1 indicated a consistent pattern within these categories. Besides, several SM showed p-values close to significance, suggesting a possible coordinated dysregulation of the sphingolipid pathway. These trends are in line with previous evidence linking sphingolipids to inflammation, insulin resistance, and lipid-related complications (60), and support the biological plausibility of our findings. Furthermore, the persistence of an altered lipid profile, particularly elevated LPCs, years after achieving SVR raises the possibility of long-term dysregulation of lipid-metabolizing enzymes such as phospholipase A2 (PLA2) (61). A plausible mechanism underlying this phenomenon is the epigenetic imprint resulting from chronic HCV infection. HCV is known to induce stable epigenetic modifications in hepatic cells, including DNA methylation and histone changes, that can persist after viral clearance (23). Such persistent epigenetic marks could influence the expression of genes involved in lipid metabolism and inflammation, thereby sustaining metabolic disturbances even in the absence of active viral replication.

Several GPs associated with metabolic events were positively correlated with the pro-inflammatory cytokine IL-18, the anti-inflammatory suppressor IL-1RA, and the ICPs IDO and S100A8/A9. Mechanistically, the correlation between LPCs and IL-18 observed in our study supports the hypothesis of inflammasome activation. LPCs are known to stimulate the NLRP3 inflammasome (62), resulting in Caspase-1 activation and subsequent cleavage and release of mature IL-18 (63). Consequently, the accumulation of LPCs may perpetuate chronic inflammation via the inflammasome pathway, contributing to the pathogenesis of metabolic disorders in these patients. This supports an inflammatory and immune activation state in participants who will develop metabolic events characterized by increased levels of pro-inflammatory factors – and also anti-inflammatory factors to counteract inflammation – and activated circulating immune cells. IL-18 is a potent pro-inflammatory cytokine involved in host defense against infections and regulates the innate and acquired immune response (64). Specifically, IL-18 is increased in viral infections and plays a central role in viral clearance by activating CD8+ T cells (65). In addition, increased plasma levels of IL-18 have been associated with various metabolic parameters, such as hepatic enzymes, lipid profiles, estimates of insulin resistance, and cardiometabolic syndrome in uninfected participants (65). In particular, IL-18 has been reported to be increased in different cohorts of participants with T2DM (66–69), which is concordant with our findings. On the other hand, IL1-RA is a secreted anti-inflammatory cytokine that blocks the binding of active pro-inflammatory IL-1 (70). It could be upregulated to protect from uncontrolled systemic inflammation in these participants.

Regarding ICPs, IDO is upregulated in HCV infection and is induced by pro-inflammatory cytokines and activated T cells. IDO up-regulation is an efficient strategy used by the virus to escape T-cell immunity as tryptophan depletion and the resulting metabolites of the catabolized IDO reaction induce an immunosuppressive environment through provoking tolerogenicity of antigen-presenting cells (APCs), immune cell death, and T-cell apoptosis (71, 72). Plasma IDO is increased in HCV-related liver cirrhosis and HCV-related hepatocellular carcinoma compared to other HCV participants and healthy controls (71). Similarly, it has been associated with hepatocyte necrosis and intrahepatic inflammation and could, therefore, potentially be used as an index of disease severity and degeneration in HCV patients (71). Hence, IDO inhibitors have been proposed to regulate metabolic disorders in patients with cardiometabolic diseases (73). Additionally, S100A8/A9 is derived from immunocytes such as macrophages and neutrophils, modulating inflammation by stimulating leukocyte recruitment and inducing cytokine secretion (74, 75). Several studies have reported that S100A8/A9 has potential as a predictive biomarker in several inflammation-associated diseases such as rheumatism, inflammatory bowel disease, obesity, and atherosclerosis, among others (76, 77). In relation to insulin resistance and T2DM, results are conflicting; some studies did not find any correlation between S100A8/A9 and markers of glucose metabolism (78), whereas other studies found S100A8/A9 to be correlated to insulin resistance (79, 80) and T2DM (81). To our knowledge, it is the first time associating IDO and S100A8/A9 with metabolic disturbances in HIV/HCV-coinfected participants.

This study has several limitations. First, the sample size is limited, although it reached the required number of samples based on MetSizeR (82), a software designed to estimate the number of samples necessary for metabolomic experiments with two groups. Second, different HCV therapies (IFN-based therapy and DAAs) could have biased the results, although we controlled for this factor by including it as a covariate in the GLM analysis.

Our study has several strengths. First, participants with hepatitis B virus (HBV) coinfection or hepatic decompensation were excluded, which provided high homogeneity to the analysis. Second, this study provides novel and valuable information, as no lipidomic analysis investigating its association with metabolic events years later in HCV/HIV-coinfected participants has been previously described.

In conclusion, specific lipid and immune dysregulation before HCV treatment was associated with the development of metabolic events (T2DM and/or hyperlipidemia) after HCV eradication in HIV/HCV-coinfected participants, suggesting that HCV leaves an aberrant signature on lipid metabolism that may potentially contribute to more severe pathologies after viral clearance.

## Data Availability

The raw data supporting the conclusions of this article will be made available by the authors, without undue reservation.

## Supplementary material

Supplementary Data Sheet 1 Members of the GeSIDA 10318 Study Group.

Supplementary Data Sheet 2 Additional description of the methods section.

Supplementary Figure 1 Multivariate analysis for the occurrence of metabolic events after HCV eradication among HIV/HCV-coinfected participants.

Supplementary Figure 2 Violin plots showing the baseline intensity levels (log10-transformed) of the most significant lipid species associated with the development of metabolic events. Data points represent individual participants, categorized by those who developed metabolic events (blue) versus those who did not (salmon). Horizontal lines represent the median.

Supplementary Table 1 Extended data for significant associations of lipids at baseline with the occurrence of metabolic events during the follow-up time in HIV/HCV-coinfected participants.Figure legends.
